# Effects of Branched-Chain Amino Acids on Parameters Evaluating Sarcopenia in Liver Cirrhosis: Systematic Review and Meta-Analysis

**DOI:** 10.3389/fnut.2022.749969

**Published:** 2022-01-27

**Authors:** Abdulrahman Ismaiel, Camelia Bucsa, Andreea Farcas, Daniel-Corneliu Leucuta, Stefan-Lucian Popa, Dan L. Dumitrascu

**Affiliations:** ^1^2nd Department of Internal Medicine, “Iuliu Hatieganu” University of Medicine and Pharmacy, Cluj-Napoca, Romania; ^2^Drug Information Research Center, “Iuliu Hatieganu” University of Medicine and Pharmacy, Cluj-Napoca, Romania; ^3^Department of Medical Informatics and Biostatistics, “Iuliu Hatieganu” University of Medicine and Pharmacy, Cluj-Napoca, Romania

**Keywords:** branched-chain amino acids (BCAA), sarcopenia, liver cirrhosis, anthropometric parameters, skeletal muscle index (SMI), mid-arm muscle circumference (MAMC), systematic review, meta-analysis

## Abstract

**Introduction:**

Sarcopenia is a major element of malnutrition in liver cirrhosis (LC) and is present in 30–70% of this population, being associated with a poor overall prognosis due to related complications such as hepatic encephalopathy, ascites, and portal hypertension. This systematic review and meta-analysis aimed to evaluate the effects of branched-chain amino acids (BCAA) supplementation on several parameters used to assess sarcopenia in LC.

**Materials and Methods:**

A comprehensive systematic electronic search was performed in PubMed, EMBASE, Scopus, Cochrane Library, and ClinicalTrials.gov databases using predefined keywords. We included full articles that satisfied the inclusion and exclusion criteria. Quality assessment of included studies was conducted using Cochrane Collaboration's tool and NHLBI quality assessment tools for interventional and observational studies, respectively. The principal summary outcome was the mean difference (MD) in the evaluated parameters. We performed a pre- and post-intervention analysis and comparison between two intervention groups (BCAA vs. controls) of the evaluated parameters when applicable.

**Results:**

A total of 12 studies involving 1,225 subjects were included in our qualitative synthesis and five in our quantitative synthesis. At baseline vs. post-intervention assessment, subjects receiving BCAA supplementation were found to have a significant improvement in skeletal muscle index (SMI) (−0.347 [95% CI −0.628–0.067; *p*-value 0.015]) and mid-arm muscle circumference (MAMC) (−1.273 [95% CI (−2.251–0.294; *p*-value 0.011]). However, no improvements were reported in handgrip (-0.616 [95% CI −2.818–1.586; *p*-value 0.584]) and triceps subcutaneous fat (1.10 [95% CI −0.814–3.014; *p*-value 0.263]).

**Conclusion:**

Following BCAA supplementation, several parameters used to evaluate sarcopenia in LC patients were found to be improved, including SMI and MAMC. Nevertheless, no improvements were seen in handgrip and triceps subcutaneous fat. Results should be interpreted with caution due to the limited methodological quality of the included studies.

## Introduction

Sarcopenia is a syndrome proposed by Rosenberg in 1989, defined as an age-related muscle mass reduction and abnormalities in muscle function, including muscle strength and physical performance (1, 2). Sarcopenia can be categorized as primary when associated with aging or secondary when related to an underlying condition such as systemic diseases, including chronic liver disease (CLD), being one of the main causes of secondary sarcopenia (3, 4).

Sarcopenia is a major element of malnutrition in liver cirrhosis and has been reported to be prevalent in 30–70% of this population (5). Several causes lead to the development of sarcopenia in patients with cirrhosis. These include malabsorption, dysregulated metabolism, reduced nutritional intake, hormonal alterations, increased loss of muscle, and hyperammonemia (6, 7). The overall prognosis in patients with cirrhosis is affected by sarcopenia due to other related complications such as hepatic encephalopathy, ascites, and portal hypertension (8, 9). The importance of sarcopenia is that it is associated with reduced quality of life, survival, mobility, and cardiopulmonary performance, as well as unfavorable metabolic outcomes and increased infection rates when compared to non-sarcopenic patients (3, 4).

**Figure 1:**
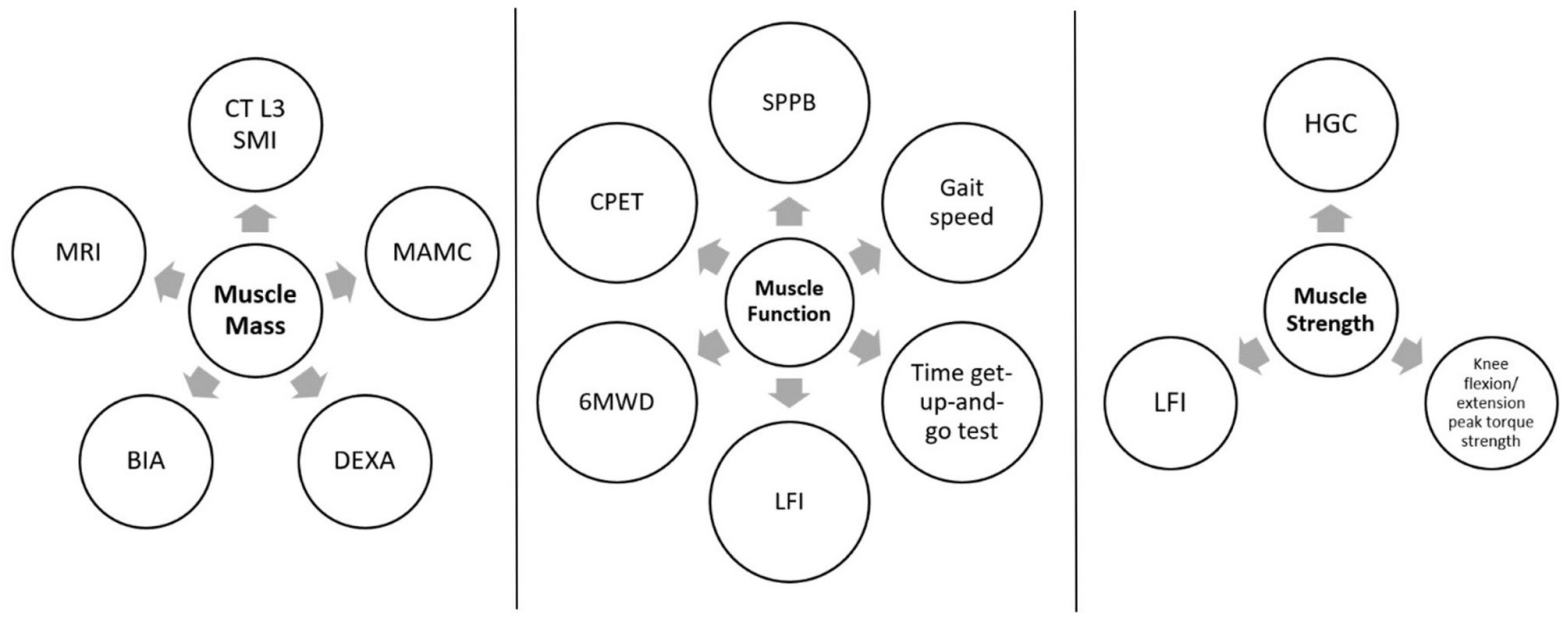
Tests used to evaluate sarcopenia, assessing muscle mass, function, and strength. CT, computed tomography; SMI, skeletal muscle index; MRI, magnetic resonance imaging; MAMC, mid-arm muscle circumference; DEXA, dual-energy X-ray absorptiometry; BIA, bioimpedance analysis; SPPB, short physical performance battery test; LFI, Liver Frailty Index; 6MWD, 6-minute walk distance; CPET, cardiopulmonary exercise testing; HGC, Handgrip strength.

Most studies evaluating sarcopenia were conducted in community-dwelling elderly patients for whom several consensus definitions have been published (3, 4, 10). Nevertheless, applying the existing consensus definition in liver cirrhosis patients is challenging due to muscle mass changes that develop in this population influencing the measurements, possibly due to altered hepatic function and water retention in peripheral edema and ascites. Furthermore, a clear consensus defining sarcopenia in liver cirrhosis patients remains required. Several tests have been used to assess sarcopenia in cirrhosis (11). Figure 1 summarizes the frequently used tests to evaluate sarcopenia, including muscle mass, function, and strength evaluation (11).

Although the clinical significance of sarcopenia in cirrhosis has been widely recognized, effective therapies are still to be discovered, mainly due to the limited available data describing the mechanisms relating sarcopenia to cirrhosis, a condition believed to be associated with a state of anabolic resistance (12, 13). Studied therapeutic approaches include diets rich in protein and fiber, nutrients supplementation with branched-chain amino acids (BCAAs), minerals, and vitamins, as well as exercise (14–16).

The concentration of BCAAs in plasma and skeletal muscle are reduced in cirrhosis (17, 18). BCAAs have been shown to be helpful as a nutritional supplement in liver cirrhosis (19–21). Several clinical trials reported the efficacy of BCAAs for nutritional status, general status, hepatic encephalopathy, and quality of life (22–25). Therefore, it is expected that BCAA supplementation may be considered a useful therapeutic modality in treating decreased muscle mass and strength that accompany secondary sarcopenia.

This comprehensive systematic review and meta-analysis aimed to investigate the effects of BCAA supplementation in observational and interventional studies on several parameters used to assess sarcopenia in patients with liver cirrhosis, including muscle mass, function, and strength evaluation such as mid-arm muscle circumference (MAMC) and skeletal muscle index (SMI), as well as handgrip strength and triceps subcutaneous fat assessment.

## Methods

This systematic review and meta-analysis was written according to the Preferred Reporting Items for Systematic Reviews and Meta-Analyses (PRISMA) checklist 2020 (26).

### Data Sources and Search Strategy

We aimed to review the currently available evidence published on PubMed, EMBASE, Scopus, Cochrane Library, and ClinicalTrials.gov, trying to answer the PICOS research question: identifying the population of patients with liver cirrhosis, with a BCAA supplementation intervention, vs. a comparator (maltodextrin), or a before after design, to observe several parameters assessing sarcopenia, as outcomes, including SMI and MAMC, as well as handgrip strength and triceps subcutaneous fat, in interventional or observational studies. A description of the conducted search strategy is provided in Supplementary Material 1. Moreover, we performed a manual search for missed publications by screening the references of included articles to minimize the risk of missing relevant studies. The conducted search included published articles from inception up to 14 July 2021. No search filters or restrictions were applied in regards to duration, country, or language. Afterward, a screening assessment was conducted by evaluating titles and abstracts for appropriateness. Articles that were selected based on the inclusion and exclusion criteria were evaluated through a full-text review. Eligibility of the evaluated studies was performed independently by two authors (A.I. and D.C.L.), and data extraction from eligible studies was performed by two authors (C.B. and A.F.), while resolving any discrepancies by mutual consensus.

### Eligibility Criteria

Inclusion criteria of original articles were as follows: (1) Full article interventional (clinical trials, RCTs) or observational studies (observational cohort population-based/ hospital-based, cross-sectional or case-control designs), evaluating the effects of BCAA supplementation on anthropometric and functional parameters assessing sarcopenia in patients with cirrhosis; (2) Liver cirrhosis evaluated using liver biopsy or imaging techniques such as ultrasonography, computed tomography (CT), magnetic resonance imaging (MRI), codes such as International Classification of Diseases (ICD), or as per study definition; (3) Parameters assessing sarcopenia according to each studies definition; (4) Human studies only; and (5) Studies published in English, French, German or Romanian languages.

Exclusion criteria were as follows: (1) Subjects with end-stage liver disease who received a liver transplant; (2) Presence of hepatocellular carcinoma; (3) Editorials, letters to the editor, case reports, conference abstracts, literature, and systematic reviews, practice guidelines, commentaries, clinical trial registrations, abstracts published without a full-text article; and (4) Experimental studies.

### Risk of Bias Assessment in Individual Studies

The risk of bias for randomized controlled trials was assessed using the Cochrane Collaboration's tool (27). The quality assessment was based on randomized sequence generation, treatment allocation concealment, blinding, and completeness of the outcome data, in addition to selective outcome reporting and other sources of bias.

For non-RCT studies, we used the National Heart, Lung, and Blood Institute (NHLBI) quality assessment tools (28). Two NHLBI tools were used, the Quality Assessment Tool for Observational Cohort and Cross-Sectional Studies, as well as the Quality Assessment Tool for Before-After (Pre-Post) Studies with No Control Group.

We used these evaluation tools to evaluate bias risk and internal validity in individual studies in a similar manner. The risk of bias in individual studies was evaluated by two authors independently (A.I. and D.C.L.). In case of disagreement, a consensus was reached through a discussion.

### Summary Measures and Synthesis of Results

The principal summary outcome was the mean difference (MD) of several parameters, including SMI, MAMC, handgrip, and triceps subcutaneous fat. For the summary outcomes, we computed the estimates of the random effects using restricted maximum likelihood to estimate the heterogeneity variance, since we assumed clinical variability between the studies. We conducted the data analyses of the meta-analysis using R with Metafor package (OpenMeta [Analyst]) (29, 30). Between-study heterogeneity was evaluated using the χ^2^ based Q-test and *I*^2^. According to the recommendations of the Cochrane Handbook (31) for identifying and measuring heterogeneity, we estimated *I*^2^ values of 0% to 40% as not important; 30% to 60% as moderate heterogeneity; 50–90% as substantial heterogeneity; and 75% to 100% as considerable heterogeneity.

The estimated total effect size analysis was calculated using the random-effects model and MD. In studies that reported medians and interquartile ranges, we calculated the mean and standard deviation (SD) to perform statistical analyses of the obtained data (32). In studies reporting results at baseline and post-intervention data, the mean change and SD change were used if they were reported. Still, in case they were not reported, they were calculated based on the before and after values according to the Cochrane Handbook recommendation using the correlation coefficient from the same study or imputed from a similar study (27). Data was reported from each study as the estimated MD with a 95% confidence interval (CI). A statistically significant *p*-value was considered when <0.05. The analyses were conducted if two or more studies evaluated similar groups and reported the same outcome using mean +/– SD or median (IQR). We also performed baseline and post-intervention analysis when available in single studies. For baseline and post-intervention analysis, we only included groups that received solely BCAA supplementation. We were not able to perform publication bias assessment due to the limited number of included studies.

## Results

### General Results

**Figure 2:**
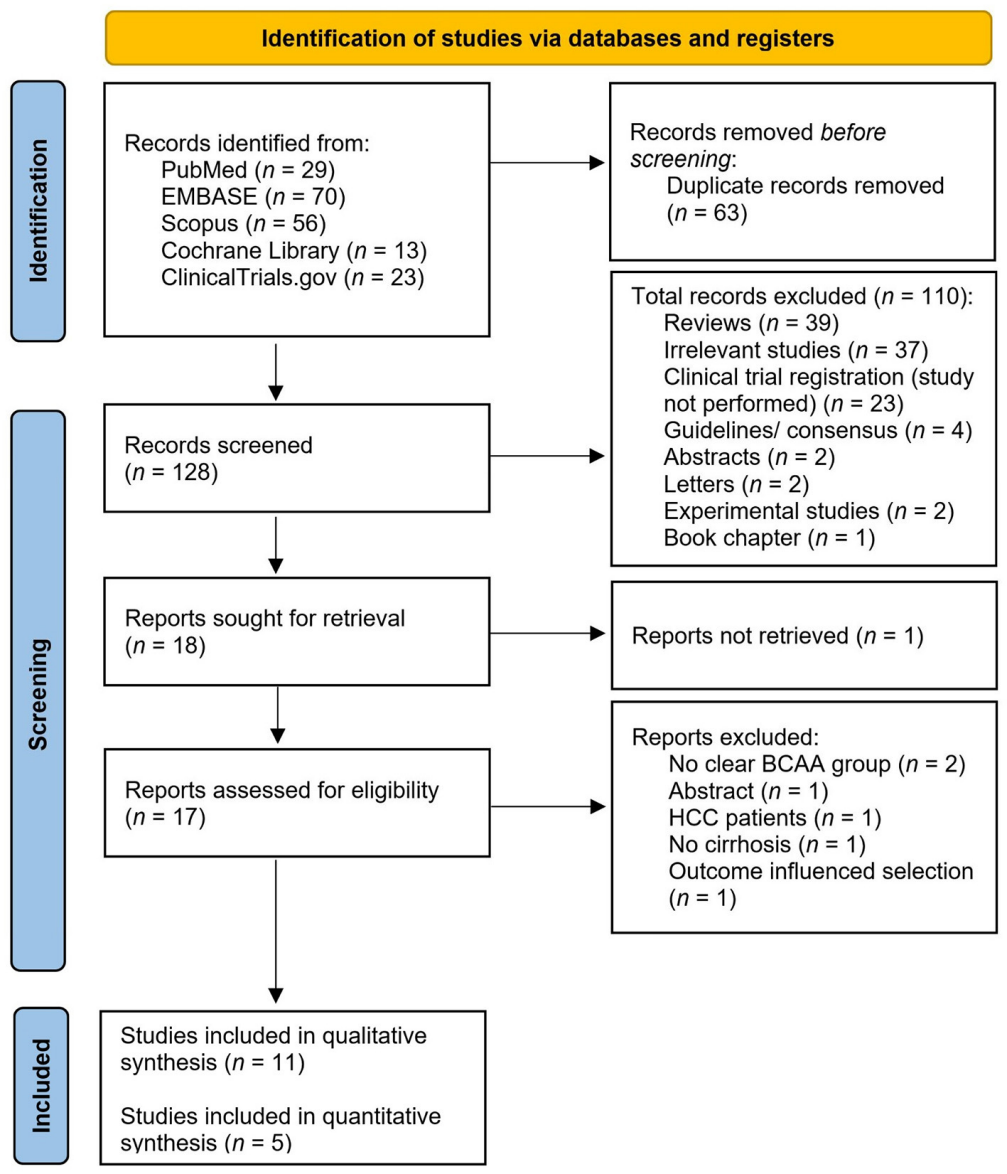
PRISMA flow diagram describing the identification, screening, and inclusion phases.

Figure 2 outlines the PRISMA flow diagram describing the performed search strategy. The initial search yielded 191 articles (PubMed *n* = 29, EMBASE *n* = 70, Scopus *n* = 56 articles, ClinicalTrials.gov *n* = 23 articles, Cochrane Library *n* = 13 articles). A total of 63 studies were removed after being detected as duplicates. After excluding the duplicates, 128 articles underwent a preliminary screening by assessing the title and abstract for inclusion and exclusion criteria fulfillment. During the screening phase we excluded 110 articles. Eighteen articles were sought for retrieval, out of which the full text of one article was not found (we contacted the authors by email, but we didn't receive any feedback). We performed a thorough reading and evaluation of the full texts for further eligibility assessment of the remaining 17 articles. We excluded six out of these articles with reasons as follows: (1) no clear BCAA group (33, 34), (2) abstract without full text (35), (3) involving hepatocellular carcinoma (HCC) patients (36), (4) not involving cirrhosis patients (37), and (5) outcome influenced selection (38). Accordingly, the qualitative synthesis included 11 articles, out of which five were included in the quantitative synthesis (38–49).

### Study Characteristics

The main characteristics of included studies are summarized in Table 1. This systematic review and meta-analysis included a total number of 1,215 individuals (394 individuals in RCTs, 821 individuals in observational studies).

**Table 1:** Characteristics of studies included in the systematic review and meta-analysis.

**No**.	**Study, setting**	**Study type**	**Population included**	**BCAA/BCAA + Intervention**	**Comparison**	**Number of patients included**	**Study participant's characteristics**	**Measurements**	**Main findings**
1	Marchesini et al., 2003; Setting: Europe	RCT, double-blind, multicenter	Patients with liver cirrhosis, portal hypertension, with Child–Pugh score ≥7	BCAA (14.4 g/day composed of 1.2 g L-leucine, 0.6 g L-isoleucine, and 0.6 g L-valine, 225 Kcal/day) for 12 months	Lactoalbumin OR Maltodextrins for 12 months	*n* = 59 BCAA group/ 56 Lactoalbumin group/*n* = 59 Maltodextrins group	59 Y in BCAA group/60 years in Lactoalbumin group/59 Y in Maltodextrins group; 36.8% F	Midarm circumference (to the nearest millimeter) using a tape meter; skinfold thickness using a caliper	Significant ↑in triceps skinfold thickness and midarm fat area in BCAA group
2	Les et al., 2011; Setting: Spain	RCT, double-blind, multicenter	18–85 years, with liver cirrhosis and hospitalized for hepatic encephalopathy, compliant with a standard diet 2 weeks before inclusion	BCAA (30 g/day, 120 Kcal/day) composed of leucine: 13.5 g, isoleucine: 9 g, valine: 7.5 g or MDX, for 56 weeks	Maltodextrin	*n* = 58 BCAA group/*n* = 58 Maltodextrin group	64.1 Y in BCAA group/62.5 Y in Maltodextrin group; 24% F	Midarm muscle circumference; muscular strength by handgrip (methods of measurements not provided)	Midarm muscle circumference and handgrip ↑in patients in the BCAA group
3	Hanai et al., 2015; Setting: Japan	Retrospective cohort study	>18 years with liver cirrhosis	^a^BCAA 12 g/day, 4 g of BCAA per sachet composed of 952 mg L-isoleucine, 1904 mg L-leucine, and 1144 mg L-valine, for >12 months	NA	*n* = 94 BCAA group/*n* = 36 non-BCAA group	66 Y in BCAA group/64 Y in non-BCAA group; 42% F	SMI using CT scan	A possible association between BCAA administration and ↑outcome in four sarcopenic patients with liver cirrhosis was observed
4	Tsien et al., 2015; Setting: Cleveland, USA	Prospective study	Patients with alcoholic cirrhosis, with Child–Pugh score ≤ 7 and healthy controls	Leucine enriched BCAA, 15 g composed of BCAA/LEU (7.5 g L-leucine, 3.75 g L-isoleucine, 3.75 g L-valine), single administration in patients with cirrhosis	Leucine enriched BCAA, 15 g single administration in healthy controls	*n* = 6 patients with cirrhosis/*n* = 8 healthy controls	54 Y in patients with cirrhosis group/45 Y in healthy controls; 35.7% F	Body composition characteristics assessed using dual-energy Xray absorptiometry. Muscle expression of myostatin, mTOR targets, autophagy markers, protein ubiquitination and intracellular amino acid deficiency sensor by muscle biopsy	Impaired mTOR1 signaling and ↑autophagy in skeletal muscle of alcoholic cirrhosis patients is acutely reversed by leucine enriched BCAA
5	Hiraoka et al., 2017; Setting: Japan	Observational, prospective (pre-post intervention)	Patients with liver cirrhosis	BCAA (protein 13.5 g, 210 kcal/day including L-leucine 1922.5 mg) + walking exercise (additional 2,000 steps daily)	NA	*n* = 33 (one group)	67 Y; 60.6% F	Muscle volume using bioelectrical impedance; handgrip strength using a hand dynamometer; leg strength using a position controllable cycle ergometer	BCAA supplementation and walking exercise were found to be effective and easily implemented for ↑muscle volume and strength in liver cirrhosis patients
6	Uojima et al., 2017; Setting: Japan	Single-center, prospective study (pre-post intervention)	Patients >20 years, with liver cirrhosis, with albumin level <3.5 g/dl after standard nutrition therapy for at least 28 days	BCAA (2*50 g/day, 420 Kcal/day), one package of BCAA (50 g) was composed of 13.5 g of protein, including L-leucine, L-isoleucine, and L-valine, which provided 210 kcal of energy; for 24 weeks	NA	*n* = 82 (one group)	69 Y; 44% F	SMI using bioelectrical impedance analysis; hand grip using a grip dynamometer	BCAA supplementation ↑low muscle strength in patients with chronic liver disease, but did not increase muscle mass during the treatment period
7	Kitajima et al., 2017; Setting: Japan	Observational, prospective; (pre-post intervention)	Patients with liver cirrhosis	BCAA granules (25–35 Kcal/kg/day and protein intake to 1.0–1.4 kg/day), each packet of BCAA contained 952 mg L-isoleucine, 1904 mg L-leucine, and 1144 mg L-valine; for 48 weeks	NA	*n* = 21 (one group)	71.3 Y; 57.1% F	Skeletal muscle volume using CT scan and bioelectrical impedance analysis; intramuscular adipose tissue content using CT scan	BCAA were associated with ↑albumin levels in patients with hypoalbuminemia and were related to maintained skeletal muscle mass
8	Ruiz-Margáin et al., 2018; Setting: Mexico	RCT, open-label	Patients 18–65 years, with liver cirrhosis	BCAA (110 g/day) + High-protein, High-fiber diet, composed of 3.38 g of L-leucine, 2.75 g of L-isoleucine, and 2.5 g of L-valine, totaling 500 Kcal for 6 months	High-protein, High-fiber diet	*n* = 37 BCAA group/*n* = 35 control group	54.9 Y in BCAA group/47.8 years in Control group; 80.6% F	Triceps skinfold thickness and mid-arm muscle circumference	↑in muscle mass and a decrease in fat mass in the BCAA group, but not in the control group
9	Hiraoka et al., 2019; Setting: Japan	Observational, prospective (pre-post intervention)	Patients with liver cirrhosis and BCAA supplementation (12.45 g/day)	Levocarnitine (1000 mg/day) + exercise (plus 2000 steps/day), for 6 months	NA	*n* = 18 (one group)	68.4 Y, 44.4% F	Muscle volume using bioelectrical impedance analysis; hand grip using a grip dynamometer; Leg muscle strength using position controllable cycle ergometer	No significant changes in the ratios of handgrip strength, leg strength, and muscle volume after 6 months
10	Hanai et al., 2020; Setting: Japan	Observational, retrospective cohort study	Patients >20 years, with liver cirrhosis	^a^BCAA (6.1 g)-enriched powder mix daily use composed of 1.602 g of L-valine, 2.037 g of L-leucine, and 1.923 g of L-isoleucine (213 kcal)	No administration	*n* = 87 BCAA group/*n* = 436 No-BCAA group	69 Y BCAA group/66 years No-BCAA group; 54.7% F	SMI	Nocturnal BCAA supplementation was associated with a significant ↓in the risk of death
11	Okubo et al., 2021; Setting: Japan	RCT, open-label	≥20 years, with decompensated cirrhosis and treated with BCAA for at least 6 months	Vitamin D, 2000 IU for 12 months	No administration	*n* = 15 Vitamin D group/*n* = 17 Control group	73 Y Vitamin D group/70 years Control group; 59.4% F	Grip strength using a grip force meter; skeletal muscle volume using bioelectrical impedance analysis	In Vitamin D group: SMI values significantly ↑; median change rates in the SMI were +5.8%; prevalence of sarcopenia significantly ↓from 80% to 33%

*BCAA, branched-chain amino acid; SMI, skeletal muscle index; SMA, skeletal muscle area; CT, computed tomography; F, females; NA, not applicable; RCT, randomized-controlled trial; Y, years; ↑, increased; ↓, decreased;*

a *non-interventional retrospective studies where BCAA were previously administered according to current guideline in Japan*.

Four studies had an interventional study design (39, 40, 46, 49), five studies had a prospective study design (42–45, 47), and two had a retrospective design (41, 48). Seven studies were undertaken in Asia (Japan *n* = 7), two in Europe (multicenter *n* = 1, Spain *n* = 1), and two in the Americas (USA *n* = 1, Mexico *n* = 1).

### Effects of BCAA Supplementation on Sarcopenia in Patients With Cirrhosis

Several parameters were evaluated in the included studies, assessing the effects of BCAA supplementation in sarcopenic patients with liver cirrhosis, including muscle mass, function, and strength, as demonstrated in Supplementary Table 1. These parameters included MAMC, SMI, skeletal muscle area, handgrip strength, tricipital skinfold thickness, bicipital skinfold thickness, suprailiac skinfold thickness, subscapular skinfold thickness, midarm muscle area, midarm fat area, fat mass, fat-free mass.

#### Skeletal Muscle Index Improvement (Baseline and Post-intervention)

**Figure 3:**
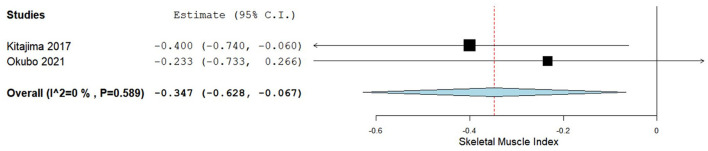
Skeletal muscle index difference between baseline and post-BCAA supplementation groups.

A total of two studies reported mean +/– SD or median (IQR) for the SMI (cm^2^/m^2^) involving baseline values and post-BCAA supplementation (45, 49). Figure 3 summarizes the obtained results regarding SMI, which was evaluated using CT scan and bioelectrical impedance analysis.

The pooled analysis assessing the SMI in baseline minus post-BCAA supplementation groups demonstrated that SMI significantly increased after the BCAA supplementation, the difference being of −0.347 (95% CI −0.628−0.067), *p*-value of 0.015. Very low heterogeneity was reported with an *I*^2^ = 0% and *p*-value 0.589.

#### Mid-Arm Muscle Circumference Post-intervention (BCAA vs. M-DXT)

**Figure 4:**
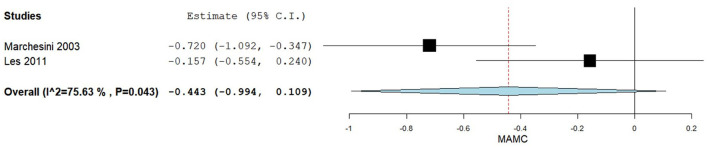
Mid-arm muscle circumference post-intervention in BCAA group vs. M-DXT group.

A total of two studies reported mean +/– SD for the MAMC (cm) comparing BCAA group vs. maltodextrins (M-DXT) group (39, 40). Figure 4 summarizes the obtained results regarding MAMC.

In the pooled analysis assessing the MAMC in BCAA group vs. M-DXT group, we observed overall larger MAMC post-intervention values in the M-DXT group compared to BCAA group, but they did not reach the statistically significant threshold, the mean difference between the groups being−0.443 (95% CI −0.994–0.240), *p*-value of 0.116. Substantial heterogeneity was reported with an *I*^2^ = 75.63% and *p*-value 0.043.

#### Mid-Arm Muscle Circumference Improvement (Baseline and Post-intervention)

**Figure 5:**
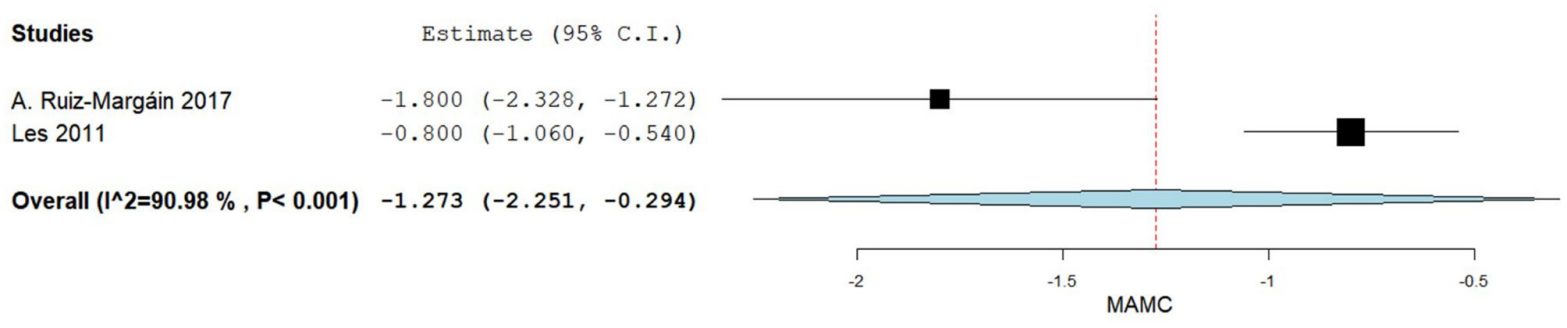
Mid-arm muscle circumference difference between baseline and post-BCAA supplementation.

A total of two studies reported mean +/– SD for the MAMC (cm) comparing baseline values and post-BCAA supplementation (40, 46). Figure 5 summarizes the obtained results regarding MAMC.

The pooled analysis assessing the difference in MAMC values between baseline and post-BCAA supplementation groups demonstrated that MAMC values significantly increased after the BCAA supplementation, the MD being of −1.273 (95% CI −2.251−0.294), *p*-value of 0.011. Substantial heterogeneity was reported with an *I*^2^ = 90.98% and *p*-value < 0.001.

#### Handgrip Change (Baseline and Post-intervention)

**Figure 6:**
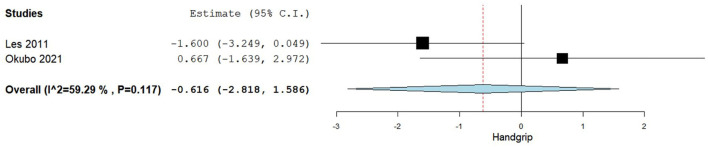
Handgrip difference between baseline and post-BCAA supplementation.

A total of two studies reported mean +/– SD or median (IQR) for handgrip (kg) comparing baseline values and post-BCAA supplementation (40, 49). Figure 6 summarizes the obtained results regarding handgrip.

In the pooled analysis assessing the difference in handgrip between baseline and post-BCAA supplementation we observed an increase in handgrip, albeit non-statistically significant, with an overall MD of −0.616 (95% CI −2.818–1.586), *p*-value of 0.584. Substantial heterogeneity was reported with an *I*^2^ = 59.29% and *p*-value 0.117.

Handgrip was evaluated in one individual study separately, comparing BCAA group vs. DXT group, assessing baseline vs. post-intervention difference. We observed a larger increase in handgrip (baseline vs. post-intervention) in BCAA group compared to DXT group, albeit non-statistically significant, with an MD of −1.0 (95% CI −2.674–0.674), *p*-value of 0.244.

#### Triceps Subcutaneous Fat Change (Baseline and Post-intervention)

Triceps subcutaneous fat was evaluated in one individual study separately, comparing BCAA group vs. controls, assessing baseline vs. post-intervention difference. We observed a non-statistically significant decrease in triceps subcutaneous fat (baseline vs. post-intervention) in BCAA group compared to controls, with an MD of 1.10 (95% CI −0.814–3.014), *p*-value of 0.263.

### Quality Assessment

Four articles were evaluated using the Cochrane Collaboration's tool (39, 40, 46, 49), five articles using the NHLBI Quality Assessment Tool for Observational Cohort and Cross-Sectional Studies (38, 41–43, 48), and three articles using the NHLBI Quality Assessment Tool for Before-After (Pre-Post) Studies with No Control Group (44, 45, 47), as demonstrated in Supplementary Tables 2–4.

Several issues were reported regarding bias in the assessed articles. Regarding RCTs evaluated in our review, although all four included RCTs had a low risk for selection bias related to random sequence generation, two of them presented an unclear risk of allocation concealment bias (46, 49). Moreover, one article presented a high risk of performance bias related to blinding of the participants and personnel as well as detection bias evaluated as outcome assessment blinding (46), while another was evaluated as unclear for both parameters being assessed (49). Incomplete outcome data, considered as attrition bias, was high in two included RCTs (40, 46). All RCTs included in this review had an unclear risk of bias regarding reporting bias and other possible sources of bias (39, 40, 46, 49).

Studies evaluated using the NHLBI quality assessment tool for observational cohort and cross-sectional studies were mainly rated as “fair” in four studies (38, 41, 43, 48) and one rated as “poor” (42). Generally, all included articles presented a clearly stated objective or research question. The study population was clearly specified and defined in three studies (38, 41, 48). The time frame was considered sufficient to reasonably expect seeing an association between BCAA supplementation and changes in parameters in only one study (41). None of the five articles evaluated using this tool clearly stated that assessors were blinded to the exposure status of the participants. Only two studies assessed potential confounding variables and performed statistical adjustments for their impact (38, 48).

A total of three studies comparing baseline with post-intervention values were evaluated using the NHLBI Quality Assessment Tool for Before-After (Pre-Post) Studies with No Control Group, with one article rated as “good” (44), one as “poor” (45), and one as “fair” (47). All three studies had a clear objective or research question, as well as clearly described eligibility criteria for the study population. All three articles used statistical methods to examine the outcome measure from before and after the intervention (44, 45, 47). Only one study did not assess the outcome measure multiple times after the underwent intervention (45).

## Discussion

Recently, the interest in sarcopenia in liver cirrhosis has increased significantly. Several published reviews evaluated the effects of interventions, including BCAA supplementation, on sarcopenia in patients with cirrhosis (11, 50, 51). However, none evaluated the effects of BCAA on anthropometric parameters assessing sarcopenia in this group population through a meta-analysis. To the best of our knowledge, this is the first systematic review and meta-analysis to evaluate the effects of supplementation with BCAAs on parameters assessing sarcopenia in liver cirrhosis. We included a total of eleven studies in our qualitative synthesis with a total population of 1,215, mainly Asian individuals, and to a lesser extent, Caucasians and Hispanics. They participated in four RCTs and seven observational studies. Furthermore, we included five studies in our quantitative synthesis, in which we demonstrated a significant improvement in SMI and MAMC parameters following BCAA supplementation, compared to non-BCAA group or baseline values, without significant improvement in handgrip and triceps subcutaneous fat. Although M-DXT was shown to slightly increase MAMC more than BCAA supplementation, no statistically significant difference was found between both groups. Moreover, handgrip was slightly better in subjects receiving BCAA supplementation compared to DXT, but without a statistically significant difference between both groups.

The new guidelines emphasize the importance of performing a functional evaluation and muscle mass quantification for evaluating sarcopenia (52). Anthropometric measures such as MAMC and triceps subcutaneous fat have been used and remain of significant importance in daily practice, reported to correlate with lean muscle mass and body fat, with an acceptable predictive value (53, 54). Nevertheless, measurement errors can occur due to lack of inter-observer agreement, as well as reduced accuracy in case of fluid overload (55). Several tools have been proposed to quantify muscle mass in clinical practice, including hand grip strength, assessed by recording the mean value of the dominant arm gripping a dynamometer in three consecutive measurements, and the chair stand test (CST), evaluated by counting the number of times the patient is able to rise fully to a standing position and subsequently sitting down in 30 seconds, without using their hands (56). Although hand grip strength was reported as an independent factor of mortality (57), on cross-sectional imaging, it was found to weakly correlated with muscle mass and quality (58). Currently, the skeletal muscle area (SMA) can be obtained using CT scan with a specific software (56). Afterwards, skeletal muscle index (SMI) in cm^2^/m^2^ can be obtained, being easily performed as the abdomen is evaluated to diagnose liver cirrhosis, while being able to discriminate between ascites and soft tissues.

Recently, researchers have attempted to find strategies that can help decrease the increased anabolic turnover rate of muscle that is age-related (59). Protein is made up of amino acids, possibly inducing a muscle protein anabolic response that is according to the availability of BCAAs, including leucine, isoleucine, and valine (37, 60). In elderly subjects, anabolic resistance and delayed absorption of amino acid absorption can be seen (61). Decreased muscle mass and function, as well as weaker muscle strength in the elderly have been associated with decreased BCAA levels (62). Moreover, aerobic exercise was found to contribute to the inductions of mitochondrial biogenesis and dynamics, mitochondrial metabolism restoration, as well as decreases the catabolic genes expression and increases muscle protein synthesis (63). Furthermore, an important strategy for preventing muscle wasting includes resistance exercise that was reported to strengthen muscle mass and function through stimulating muscle hypertrophy and improving muscle strength (64). Therefore, combining aerobic and resistance exercises can provide a greater benefit, providing a partial solution to sarcopenia. Accordingly, several studies reported that BCAA supplementation, including leucine, isoleucine, valine, or essential amino acids, in addition to aerobic and low-intensity resistance training can attenuate sarcopenia and stimulate muscle protein synthesis, even in the bedrest confined elderly subjects (37).

We believe that our results need to be further discussed. Firstly, most involved participants were from Asia, with a limited number of Caucasians and Hispanics. Moreover, ethnicities such as African Americans were not included in any of the studies evaluating parameters of sarcopenia in patients with cirrhosis. Therefore, the obtained results cannot be generalized to other ethnicities that have not been evaluated yet in the currently published evidence.

In our meta-analysis, we demonstrated that several parameters, including SMI and MAMC were improved following BCAA supplementation. Nevertheless, handgrip and triceps subcutaneous fat did not improve significantly. Although current evidence demonstrated that supplementation with BCAAs such as leucine, valine, and isoleucine could ameliorate protein synthesis, lipid, and glucose metabolism, as well as insulin resistance and hepatocyte proliferation, in addition, reduce oxidative stress in hepatocytes in liver cirrhosis, several published studies reported no significant improvement in muscle strength or mass post-intervention with BCAA supplementation (51, 65). This can be partially explained by the short intervention duration and small sample size (40, 66–68), or a specific subgroup of LC with albumin ≤ 3.5 g/dL (44, 45). Furthermore, administration timing, dose, and nutritional education regarding BCAA supplementation are also considered essential factors that might lead to suboptimal results if not properly performed. The present systematic review and meta-analysis evaluates parameters used to assess sarcopenia and did not assess sarcopenia as its presence or absence. However, it is important to assess the effects of BCAAs on these parameters, used to evaluate sarcopenia in patients with liver cirrhosis. Any findings in this respect can help gather evidence on how to treat sarcopenia as well.

According to the quality assessment of included studies in our systematic review and meta-analysis, most evaluated studies using both NHLBI quality assessment tools were rated as “poor” and “fair,” while only one study was evaluated as “good.” Moreover, half of the included RCTs presented an unclear risk of allocation concealment bias, in addition to unclear risk of reporting bias and other possible sources of bias in all included RCTs. Another point to consider is that several studies did not perform statistical adjustments for potential confounding variables. Therefore, the obtained results from studies with poor and fair methodological quality should be cautiously interpreted.

Most included studies were of observational design and not interventional. Therefore, more interventional studies are required in order to confirm the possible causality between BCAA supplementation and improvements in parameters assessing sarcopenia in patients with cirrhosis. Furthermore, we were not able to perform subgroup analysis evaluating variables according to the etiology of liver cirrhosis or administrated BCAA supplementation due to limited available data.

Our systematic review and meta-analysis has several limitations that need to be addressed. Multiple included studies in this review are of observational design. Therefore, causality between BCAA supplementation and improvement or worsening of sarcopenia assessed parameters in cirrhosis cannot be confirmed or negated according to these studies. Although several parameters assessing sarcopenia, including muscle mass, function, and strength evaluation, were conducted in the included studies in our review, most studies had a different grouping of the involved participants or interventions performed, leading to a very limited number of studies that were possibly included in our quantitative synthesis. Furthermore, due to a limited number of published articles assessing several parameters used to assess sarcopenia in liver cirrhosis patients receiving BCAA supplements, we could only assess two studies for each association. The pre-post comparisons encountered in some meta-analyses are subject to possible biases, being less desirable to classic between-arms comparisons. Short intervention intervals and small sample sizes can also lead to suboptimal results. Accordingly, future research involving larger populations with longer interventional intervals involving BCAA supplementation is deemed necessary. Due to possible methodological flaws in included studies, results should be interpreted with caution.

Nevertheless, our systematic review and meta-analysis also has several important strengths. We believe that this topic is of important clinical significance due to the increased prevalence of liver cirrhosis and secondary sarcopenia, leading to more complications and higher morbidity and mortality rates. In this review, we point out several problems in current studies that should be remediated in future studies. We also performed a comprehensive search in several electronic databases while meta-analytically summarizing the current literature regarding this topic in a non-biased manner. To the best of our knowledge, this is the first systematic review and meta-analysis to assess the effects of BCAA supplementation on sarcopenia evaluated parameters in liver cirrhosis.

## Conclusions and Future Directions

Improvements in several parameters used to assess sarcopenia in liver cirrhosis patients, including skeletal muscle index, mid-arm muscle circumference, were seen following BCAA supplementation. However, no improvements were seen in handgrip and triceps subcutaneous fat. Nonetheless, due to the imperfect methodological quality of the evaluated articles, interpretation of the obtained results should be performed with caution.

Future interventional studies, mainly better methodologically conducted RCTs, with larger sample sizes and longer interventional intervals evaluating the effects of BCAA supplementation on parameters used to assess sarcopenia in liver cirrhosis patients from different ethnicities, remains necessary. Possible improvements in quality of life, survival, mobility, and cardiopulmonary performance, in addition to reduced infection rates and favorable metabolic outcomes in liver cirrhosis patients with sarcopenia, can be obtained if future RCTs confirm our reported findings. Moreover, a clear consensus defining sarcopenia in liver cirrhosis patients is required.

## Data Availability Statement

The original contributions presented in the study are included in the article/Supplementary Material, further inquiries can be directed to the corresponding author.

## Author Contributions

D-CL and DD had the idea of the manuscript and made substantial contributions to the conception and critically revised the manuscript for important intellectual content. AI and D-CL independently applied the search strategy, performed the study selection, and risk of bias assessment. CB and AF performed the data extraction. AI drafted the manuscript. D-CL, CB, AF, S-LP, and DD contributed to the writing of the manuscript. All authors revised the final manuscript and approved the final version.

## Conflict of Interest

The authors declare that the research was conducted in the absence of any commercial or financial relationships that could be construed as a potential conflict of interest.

## Publisher's Note

All claims expressed in this article are solely those of the authors and do not necessarily represent those of their affiliated organizations, or those of the publisher, the editors and the reviewers. Any product that may be evaluated in this article, or claim that may be made by its manufacturer, is not guaranteed or endorsed by the publisher.
