# Supplementary material for: Effects of Branched-Chain Amino Acids on Parameters Evaluating Sarcopenia in Liver Cirrhosis: Systematic Review and Meta-Analysis
Source: Front Nutr. 2022 Jan 27;9:749969. doi: 10.3389/fnut.2022.749969 (PMC8828569; doi:10.3389/fnut.2022.749969)
Supplement: Supplementary file 1 [file Table_1.DOCX]

Supplementary Material

# Search Strategy

**PubMed:**

(("Sarcopenia"[MeSH Terms]) OR ("Muscular Atrophy"[MeSH Terms]) OR ("Sarcopenia"[All Fields]) OR ("Muscular Atrophy"[All Fields]) OR ("muscle mass"[All Fields])) AND (("Liver Cirrhosis"[MeSH Terms]) OR ("Liver Cirrhosis"[All Fields]) OR ("CLD"[All Fields]) OR ("chronic liver disease"[All Fields])) AND (("amino acids, branched chain"[MeSH Terms]) OR ("branched-chain amino acids"[All Fields]) OR ("BCAA"[All Fields])).

**EMBASE:**

('sarcopenia'/exp OR 'sarcopenia' OR 'muscular atrophy'/exp OR 'muscular atrophy' OR 'muscle mass'/exp OR 'muscle mass') AND ('liver cirrhosis'/exp OR 'liver cirrhosis' OR 'cld' OR 'chronic liver disease'/exp OR 'chronic liver disease') AND ('branched-chain amino acids' OR 'bcaa')

**Scopus:**

'sarcopenia' AND 'liver cirrhosis' AND 'branched-chain amino acids'

**Cochrane:**

liver cirrhosis AND Sarcopenia AND branched-chain amino acids

**ClinicalTrials.gov:**

liver cirrhosis AND Sarcopenia AND branched-chain amino acids

Extracted data included author names, publication year, study design, in addition to the study characteristics including the involved population, the administered BCAA intervention, number of participants, mean age, gender distribution, outcome measures, and main study findings. Data were filled in tables, while the final data was collated and presented in the manuscript text.
