# Supplementary material for: Effects of Branched-Chain Amino Acids on Parameters Evaluating Sarcopenia in Liver Cirrhosis: Systematic Review and Meta-Analysis
Source: Front Nutr. 2022 Jan 27;9:749969. doi: 10.3389/fnut.2022.749969 (PMC8828569; doi:10.3389/fnut.2022.749969)
Supplement: Supplementary file 2 [file Table_2.DOCX]

Supplementary Tables

**Supplementary Table 1.** Evaluation of BCAA supplementation effects on sarcopenia (muscle mass, function, and strength) in cirrhosis

| **Study** | **Evaluated Parameters** | **Evaluated Groups** | **P-value** |
| --- | --- | --- | --- |
| Marchesini et al. 2003 ([39](#_ENREF_39)) | Mid-arm muscle circumference (cm) | **BCAA**: 27.1 ± 0.5  **L-ALB**: 26.7 ± 0.6  **M-DXT**: 27.5 ± 0.6 | - |
|  | Tricipital skinfold thickness (mm) | **BCAA**: 11.2 ± 0.8  **L-ALB**: 11.2 ± 0.8  **M-DXT**: 12.0 ± 0.9 | - |
|  | Bicipital skinfold thickness (mm) | **BCAA**: 6.4 ± 0.4  **L-ALB**: 6.8 ± 0.5  **M-DXT**: 7.0 ± 0.6 | - |
|  | Suprailiac skinfold thickness (mm) | **BCAA**: 12.3 ± 0.8  **L-ALB**: 12.7 ± 1.0  **M-DXT**: 12.9 ± 0.9 | - |
|  | Subscapular skinfold thickness (mm) | **BCAA**: 13.3 ± 0.7  **L-ALB**: 14.5 ± 0.9  **M-DXT**: 14.6 ± 0.9 | - |
|  | Midarm muscle area (cm2) | **BCAA**: 45.3 ± 1.7  **L-ALB**: 43.8 ± 1.8  **M-DXT**: 45.9 ± 1.8 | - |
|  | Midarm fat area (cm2) | **BCAA**: 14.4 ± 1.2  **L-ALB**: 14.3 ± 1.2  **M-DXT**: 16.1 ± 1.5 | - |
|  | Fat mass (kg) | **BCAA**: 20.1 ± 1.4  **L-ALB**: 19.8 ± 1.6  **M-DXT**: 23.0 ± 0.9 | - |
|  | Fat-free mass (kg) | **BCAA**: 50.9 ± 1.9  **L-ALB**: 49.6 ± 1.7  **M-DXT**: 49.6 ± 1.8 | - |
| Les et al. 2011 ([40](#_ENREF_40)) | Mid-arm muscle circumference (cm) | **BCAA**: **Baseline** - 21.4 ± 3.0  **BCAA**: **Final** - 22.2 ± 3.0  **MDX**: **Baseline** - 22.1 ± 2.7  **MDX**: **Final** - 22.7 ± 3.3 | - |
|  | Handgrip (kg) | **BCAA**: **Baseline** - 20.6 ± 8.5  **BCAA**: **Final** - 21.1 ± 8.4  **MDX**: **Baseline** - 22.1 ± 8.9  **BCAA**: **Final** - 22.4 ± 8.7 | - |
| Hanai et al. 2015 ([41](#_ENREF_41)) | Skeletal muscle area (cm2) | **Men**  **Total**: 119 (23–183)  **Non**-**BCAA**: 127 (23–180)  **BCAA**: 117 (53–183)  **Women**  **Total**: 85 (49–110)  **Non**-**BCAA**: 84 (61–101)  **BCAA**: 88 (49–110) | 0.88  0.60 |
|  | Skeletal muscle index (cm^2^/m^2^) | **Men**  **Total**: 45.2 (7.2–61.7)  **Non**-**BCAA**: 44.9 (7.2–60.1)  **BCAA**: 45.4 (20.1–61.7)  **Women**  **Total**: 38.4 (23.9–50.1)  **Non**-**BCAA**: 38.7 (28.8–47.5)  **BCAA**: 38.1 (23.9–50.1) | 0.45  0.85 |
| Tsien et al. 2015 ([42](#_ENREF_42)) | Body fat (%) | **Controls**: 34.63±2.05 (25.2-41.7)  **Cirrhosis**: 33.92±4.07 (19.9-44.5) | - |
|  | Lean body mass (kg) | **Controls**: 48.49±4.12 (35.72-72.58)  **Cirrhosis**: 47.68±3.93 (35.86-61.91) | - |
|  | Legs fat mass (kg) | **Controls**: 8.63±0.66 (5.32-11.17)  **Cirrhosis**: 7.54±0.66 (5.13-9.75) | - |
|  | Lean leg mass (kg) | **Controls**: 16.74±1.43 (11.68-23.91)  **Cirrhosis**: 15.71±1.21 (12.07-17.25) | - |
|  | Trunk fat mass (kg) | **Controls**: 12.88±1.16 (9.13-18.39)  **Cirrhosis**: 13.80±2.46 (4.48-22.28) | - |
|  | Trunk lean mass (kg) | **Controls**: 22.40±1.93 (16.59-34.54)  **Cirrhosis**: 22.98±2.25 (16.71-30.64) | - |
| Hiraoka et al. 2017 ([43](#_ENREF_43)) | Changes in the ratios of:  (a) muscle volume  (b) leg strength  (c) handgrip strength | **Pre and post BCAA supplementation and walking exercise** (after 3 months) | (a) 1.0–1.013, P <0.01;  (b) 1.0–1.11, P< 0.01;  (c) 1.0–1.06, P =0.01 |
| Uojima et al. 2017 ([44](#_ENREF_44)) | Skeletal muscle index (cm^2^/m^2^) | **Pre and post BCAA supplementation:**  Univariate logistic regression analysis: OR 0.769 (95% CI 0.565–1.046) | 0.0939 |
| Kitajima et al. 2017 ([45](#_ENREF_45)) | Intramuscular adipose tissue content | **Before BCAA:** -0.11 ± 0.16  **After BCAA**: -0.12 ± 0.17 | 0.7504 |
|  | Skeletal muscle index (cm^2^/m^2^) | **Before BCAA**: 12.4 ± 2.7  **After BCAA**: 12.0 ± 2.8 | 0.0735 |
| Ruiz-Margáin et al. 2018 ([46](#_ENREF_46)) | Triceps skinfold (mm) | **Baseline BCAA group**: 21.1 ± 12.2  **Final BCAA group**: 19.6 ± 7.5  **Baseline control group**: 20.7 ± 7.3  **Final Control group**: 20.3 ± 6.9 | 0.000  0.923 |
|  | Mid-arm muscle circumference (cm) | **Baseline BCAA group**: 28.7 ± 5.3  **Final BCAA group**: 30.5 ± 4.6  **Baseline control group**: 25.6 ± 6.1  **Final Control group**: 25.9 ± 6.7 | 0.000  0.966 |
| Hiraoka et al. 2019 ([47](#_ENREF_47)) | Relative changes in ratios of handgrip and leg strength | **Pre and post BCAA supplementation**:  No significant changes demonstrated in the ratio of handgrip or leg strength at any time point. | - |
|  | Relative number and ratio of daily steps | **Pre and post BCAA supplementation** at 2, 4, and 6 months:  (a) Number of daily steps  (b) Ratio of daily steps | (a) P <0.01  (b) P= 0.029 |
|  | Relative changes in ratios of muscle volume and fat | **Pre and post BCAA supplementation**:  No significant changes demonstrated in the ratios:  (a) muscle volume  (b) fat at any time point | - |
| Hanai et al. 2020 ([48](#_ENREF_48)) | Skeletal muscle index (cm^2^/m^2^) | **No LES**: 44.1 (37.8–51.2)  **LES**: 41.9 (36.5–47.9) | p-value 0.039  SMD: 0.299 |
| Okubo et al. 2021 ([49](#_ENREF_49)) | Grip strength (kg) | **Control**: 18 (10–30)  **Vit. D supplementation**: 16 (7–25) | 0.157 |
|  | Skeletal muscle mass index (kg/m^2^) | **Control**: 6.8 (5.1–8.3)  **Vit. D supplementation**: 5.5 (4.9–7.6) | 1.10 X 10^–2^ |
|  | Fat free mass (kg) | **Control**: 43.3 (34.4–56.0)  **Vit. D supplementation**: 34.4 (31.0–56.4) | 0.212 |
|  | Percentage of body fat (%) | **Control**: 31.2 (19.3–46.5)  **Vit. D supplementation**: 33.9 (13.1–46.3) | 0.935 |
| **BCAA** – branch-chained amino acids; **L-ALB** – lactoalbumin; **LES** – Late evening snack; **M-DXT** – maltodextrin. | | | |

**Supplementary Table 2.** Cochrane Risk of Bias Tool for RCTs

| **Domain** | **Marchesini et al. 2003** | **Les et al. 2011** | **Ruiz-Margáin et al. 2018** | **Okubo et al. 2021** |
| --- | --- | --- | --- | --- |
| *Selection bias – Random sequence generation* | - | - | - | - |
| *Selection bias – Allocation concealment* | - | - | ? | ? |
| *Performance bias – Blinding (participants and personnel)* | - | - | + | ? |
| *Detection bias – Blinding (outcome assessment)* | - | - | + | ? |
| *Attrition bias – Incomplete outcome data* | - | + | + | - |
| *Reporting bias – Selective reporting* | ? | ? | ? | ? |
| *Other bias – Other sources of bias* | ? | ? | ? | ? |
| **+, high risk; -, low risk; ?, unclear risk of bias.** | | | | |

**Supplementary Table 3.** NHLBI Quality Assessment Tool for Before-After (Pre-Post) Studies with No Control Group

| **Criteria** | **Uojima et al. 2017** | **Kitajima et al. 2018** | **Hiraoka et al. 2019** |
| --- | --- | --- | --- |
| *1. Was the study question or objective clearly stated?* | Yes | Yes | Yes |
| *2. Were eligibility/selection criteria for the study population prespecified and clearly described?* | Yes | Yes | Yes |
| *3. Were the participants in the study representative of those who would be eligible for the test/service/intervention in the general or clinical population of interest?* | Yes | Yes | Yes |
| *4. Were all eligible participants that met the prespecified entry criteria enrolled?* | Yes | CD | CD |
| *5. Was the sample size sufficiently large to provide confidence in the findings?* | No | No | No |
| *6. Was the test/service/intervention clearly described and delivered consistently across the study population?* | Yes | CD | Yes |
| *7. Were the outcome measures prespecified, clearly defined, valid, reliable, and assessed consistently across all study participants?* | Yes | Yes | Yes |
| *8. Were the people assessing the outcomes blinded to the participants' exposures/interventions?* | NA | NA | NA |
| *9. Was the loss to follow-up after baseline 20% or less? Were those lost to follow-up accounted for in the analysis?* | Yes | NA | Yes |
| *10. Did the statistical methods examine changes in outcome measures from before to after the intervention? Were statistical tests done that provided p values for the pre-to-post changes?* | Yes | Yes | Yes |
| *11. Were outcome measures of interest taken multiple times before the intervention and multiple times after the intervention (i.e., did they use an interrupted time-series design)?* | Yes | No | Yes |
| *12. If the intervention was conducted at a group level (e.g., a whole hospital, a community, etc.) did the statistical analysis take into account the use of individual-level data to determine effects at the group level?* | NA | NA | NA |
| ***Rating*** | **Good** | **Poor** | **Fair** |

**Supplementary Table 4.** NHLBI Quality Assessment Tool for Observational Cohort and Cross-Sectional Studies

| **Criteria** | **Hanai et al. 2015** | **Tsein et al. 2015** | **Hanai et al. 2017** | **Hiraoka et al. 2017** | **Hanai et al. 2020** |
| --- | --- | --- | --- | --- | --- |
| *1. Was the research question or objective in this paper clearly stated?* | Yes | Yes | Yes | Yes | Yes |
| *2. Was the study population clearly specified and defined?* | Yes | No | Yes | No | Yes |
| *3. Was the participation rate of eligible persons at least 50%?* | NA | NA | NA | NA | NA |
| *4. Were all the subjects selected or recruited from the same or similar populations (including the same time period)? Were inclusion and exclusion criteria for being in the study prespecified and applied uniformly to all participants?* | Yes | CD | Yes | CD | Yes |
| *5. Was a sample size justification, power description, or variance and effect estimates provided?* | No | No | No | No | No |
| *6. For the analyses in this paper, were the exposure(s) of interest measured prior to the outcome(s) being measured?* | Yes | Yes | Yes | Yes | Yes |
| *7. Was the time frame sufficient so that one could reasonably expect to see an association between exposure and outcome if it existed?* | Yes | No | CD | No | CD |
| *8. For exposures that can vary in amount or level, did the study examine different levels of the exposure as related to the outcome (e.g., categories of exposure, or exposure measured as continuous variable)?* | NA | NA | NA | NA | NA |
| *9. Were the exposure measures (independent variables) clearly defined, valid, reliable, and implemented consistently across all study participants?* | Yes | Yes | No | Yes | CD |
| *10. Was the exposure(s) assessed more than once over time?* | NA | NA | NA | NA | NA |
| *11. Were the outcome measures (dependent variables) clearly defined, valid, reliable, and implemented consistently across all study participants?* | Yes | No | Yes | Yes | Yes |
| *12. Were the outcome assessors blinded to the exposure status of participants?* | No | CD | No | CD | No |
| *13. Was loss to follow-up after baseline 20% or less?* | NA | NA | NA | Yes | NA |
| *14. Were key potential confounding variables measured and adjusted statistically for their impact on the relationship between exposure(s) and outcome(s)?* | No | No | Yes | No | Yes |
| ***Rating*** | **Fair** | **Poor** | **Fair** | **Fair** | **Fair** |
